# Ambient temperature and exclusive breastfeeding: a nationwide cohort study in Brazil

**DOI:** 10.1093/ije/dyag205

**Published:** 2026-09-17

**Authors:** Rita de Cássia Ribeiro-Silva, Taísa Rodrigues Cortes, Laís Silva Sacramento, Lais Helena Leandro Ribeiro, Otavio T Ranzani, Paulo Cerqueira Junior, Danielson Delgado, Ismael H Silveira, Lucas Santos Vieira, Enny S Paixão, Priscila Ribas de Farias Costa, Maurício L Barreto

**Affiliations:** School of Nutrition, Federal University of Bahia (UFBA), Salvador, Brazil; Center for Data and Knowledge Integration for Health (CIDACS), Oswaldo Cruz Foundation, Salvador, Brazil; Institute of Collective Health, Federal University of Bahia (ISC/UFBA), Salvador, Brazil; Center for Data and Knowledge Integration for Health (CIDACS), Oswaldo Cruz Foundation, Salvador, Brazil; Center for Data and Knowledge Integration for Health (CIDACS), Oswaldo Cruz Foundation, Salvador, Brazil; Center for Data and Knowledge Integration for Health (CIDACS), Oswaldo Cruz Foundation, Salvador, Brazil; DataHealth Lab, Institut de Recerca Sant Pau (IR SANT PAU), Barcelona, Spain; School of Statistics (UFPA), Federal University of Pará, Belém, Brazil; Center for Data and Knowledge Integration for Health (CIDACS), Oswaldo Cruz Foundation, Salvador, Brazil; Institute of Collective Health, Federal University of Bahia (ISC/UFBA), Salvador, Brazil; Center for Data and Knowledge Integration for Health (CIDACS), Oswaldo Cruz Foundation, Salvador, Brazil; Faculty of Epidemiology and Population Health, London School of Hygiene and Tropical Medicine, London, United Kingdom; School of Nutrition, Federal University of Bahia (UFBA), Salvador, Brazil; Center for Data and Knowledge Integration for Health (CIDACS), Oswaldo Cruz Foundation, Salvador, Brazil; Center for Data and Knowledge Integration for Health (CIDACS), Oswaldo Cruz Foundation, Salvador, Brazil; Institute of Collective Health, Federal University of Bahia (ISC/UFBA), Salvador, Brazil

## Abstract

**Background:**

The climate crisis presents an emerging global challenge with potential implications for breastfeeding. We estimated the association between ambient temperatures (both heat and cold) and interruption of exclusive breastfeeding (EBF) in a nationwide cohort in Brazil.

**Methods:**

The population-based longitudinal study was conducted using data from the Food and Nutrition Surveillance System (SISVAN-Web) between 2015 and 2019. EBF was defined as the consumption of breast milk only on the day of the visit, with no intake of any other liquids or foods. Daily minimum and maximum ambient temperatures and relative humidity were derived from the Brazilian Daily Weather Gridded Data (BR-DWGD). The association between ambient temperature and time to interruption of EBF was assessed using Cox proportional hazards models combined with distributed lag non-linear models (DLNMs).

**Results:**

A total of 397 239 children aged 0 to <6 months were included in the study. Nationally, low temperatures were not significantly associated with EBF interruption (hazard ratio [HR] 1.01, 95% confidence interval [CI] 0.98–1.05), whereas heat exposure was associated with a 10% higher risk (HR 1.10, 95% CI 1.08–1.14). The increased risk associated with heat exposure was similar across infant age groups, child sex, and levels of municipal material deprivation. In contrast, we found no association between cold temperatures and EBF interruption at the national level. However, our findings suggest that cold-related effects may differ across infant age and municipal deprivation levels.

**Conclusion:**

Our findings highlight the importance of strengthening breastfeeding support during periods of elevated heat and integrating climate-sensitive approaches into maternal and child health policies.

Key Messages

- We evaluated the association between ambient temperatures (both heat and cold) and interruption of exclusive breastfeeding in a nationwide cohort in Brazil.
- We found that higher ambient temperatures were associated with increased risk of early interruption of exclusive breastfeeding in Brazil, with consistent patterns across infant age groups, sex, and socioeconomic strata.
- As global temperatures continue to rise and heatwaves become more frequent and intense, understanding how heat affects breastfeeding practices is essential for informing child health interventions.

## Introduction

Breastfeeding is essential for optimal infant growth, development, and health. The World Health Organization (WHO) recommends that breastfeeding be initiated within the first hour after birth, maintained exclusively during the first 6 months of life, and subsequently complemented with appropriate foods while continued breastfeeding extends up to 2 years of age or beyond [1]. Over the past two decades, breastfeeding indicators have improved in Brazil and several countries worldwide, supported by strengthened public health initiatives and policy interventions. Nevertheless, less than half of infants globally (48% in 2024) are exclusively breastfed for the first 6 months [2]. In Brazil, the rate of exclusive breastfeeding (EBF) among infants aged 0 to <6 months increased from 35% in 2006 to 45.8% in 2019 [3]. Despite this progress, Brazil’s current trajectory suggests that the country is unlikely to achieve the WHO/UNICEF 2030 target of at least 70% [3]. The most socioeconomically disadvantaged regions of the country, especially the Northeast and North, would require even greater increases in breastfeeding rates to reach the 2030 target [3].

The climate crisis presents an emerging global challenge with potential implications for breastfeeding [4, 5]. Growing evidence indicates that infant feeding practices are susceptible to weather-related variations [5, 6]. Nonetheless, studies investigating the influence of ambient temperature and seasonal factors on breastfeeding have reported widely varying associations in both magnitude and direction [7]. A cross-sectional study from Egypt [8] reported that EBF was more common and lasted longer during warmer months. In contrast, cohort studies conducted in Pakistan [9, 10], Israel [11, 12], and West Africa [6] reported declines in EBF prevalence or duration during periods of higher temperatures. In contrast, a cohort study in Southern Brazil found that season of birth and lower ambient temperatures in the first month of life were associated with a decrease in breastfeeding duration [13].

Despite the growing evidence, prior research frequently relies on seasonal indicators (e.g. summer and winter months) instead of direct temperature measurements and shows limited generalizability across diverse climatic and socioeconomic contexts [7]. The present study aims to address these gaps in the literature by evaluating the association between ambient temperatures (both heat and cold) and interruption of EBF in a nationwide cohort in Brazil. Furthermore, we investigated disparities in the temperature–breastfeeding relationship according to infant characteristics and socioeconomic factors.

## Methods

### Data sources and study population

The present population-based longitudinal study was conducted using data from the Food and Nutrition Surveillance System (Sistema de Vigilância Alimentar e Nutricional—SISVAN-Web) between 2015 and 2019. Since its establishment in 2008, SISVAN has tracked the nutritional status of the Brazilian population attending public primary care services throughout the life course [14–16]. The primary purpose of SISVAN is to support the evaluation and formulation of public nutrition policies in Brazil. From 2015 to 2019, SISVAN recording coverage for food consumption markers increased across all life-course stages. Among children under 2 years of age, coverage increased from 0.71% to 1.12% [16].

**Figure 1:**
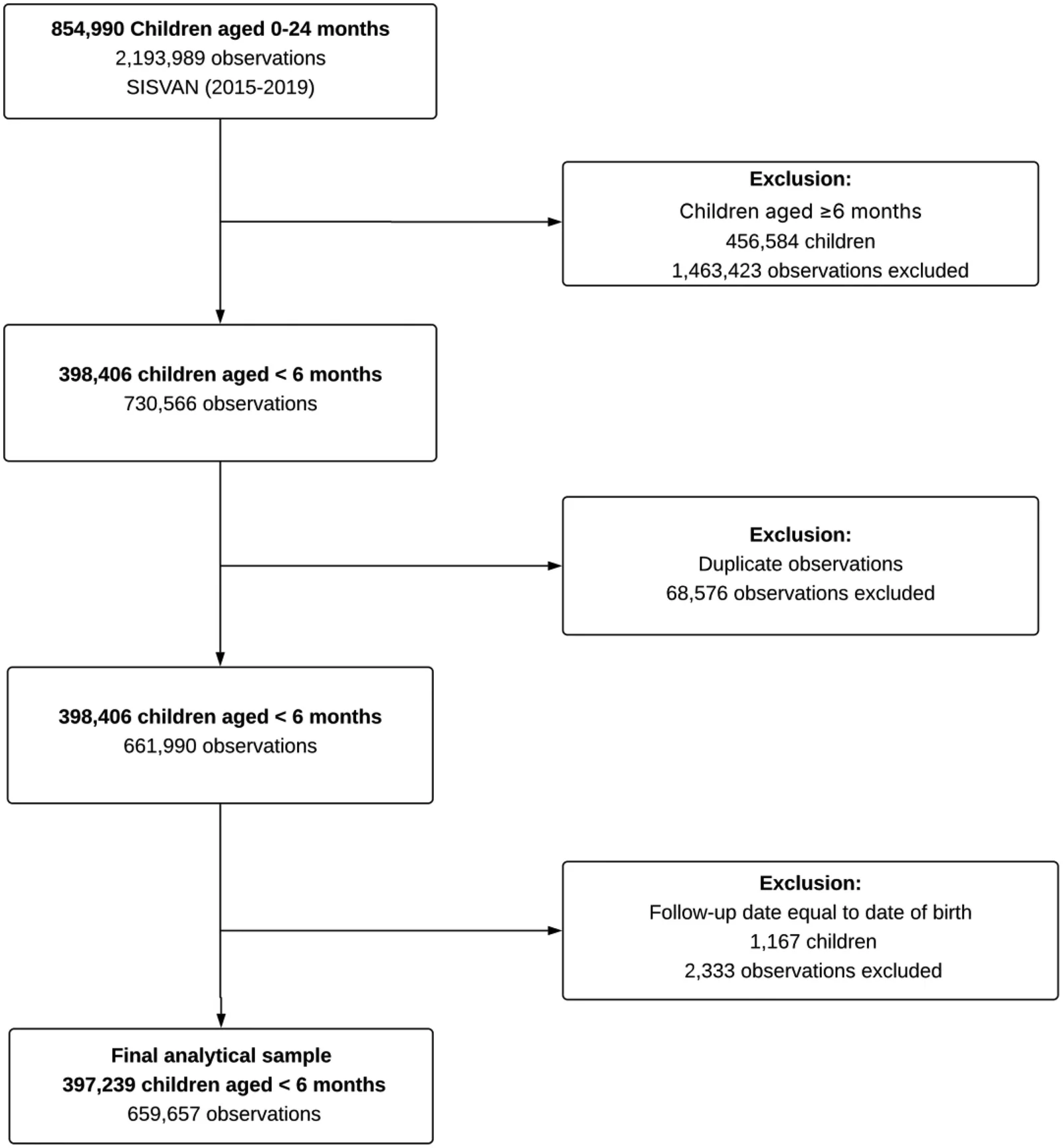
Flowchart of the cohort and relevant exclusion criteria.

A total of 854 990 children aged 0–24 months (2 193 989 observations) had nutritional monitoring records registered in SISVAN between 2015 and 2019. The final analytical sample consisted of 397 239 children aged 0 to <6 months (659 657 observations), from all 5570 Brazilian municipalities (Fig. 1).

### Outcome

Food consumption was assessed using a standardized form consisting of specific questions designed to capture qualitative information. [17]. Its validity was confirmed in a study conducted by Guedes *et al*. [18]. During routine care, primary healthcare professionals administer the questionnaire and record the caregiver’s responses as “yes,” “no,” or “don’t know.” In the present study, responses marked as “don’t know” were considered missing data. EBF was defined according to the WHO definition [19] as the consumption of breast milk only on the day before the visit, with no intake of any other liquids or foods.

### Exposure

Daily minimum and maximum ambient temperatures and relative humidity were derived from the Brazilian Daily Weather Gridded Data (BR-DWGD), which provides nationwide coverage at a high spatial resolution (0.1° × 0.1°) by interpolating data from 1252 meteorological stations [20]. Daily mean temperature was calculated as the average of daily minimum and maximum temperatures. Absolute humidity was estimated from daily mean temperature and relative humidity using standard meteorological equations [21].

Municipal-level time series were generated as the area-weighted average of grid cells intersecting each municipality’s boundaries. Finally, ambient temperature and absolute humidity data were linked to nutritional records based on the date and municipality of each child’s assessment.

### Potential confounders and effect modifiers

Potential confounders were identified based on prior literature on determinants of EBF [7, 22, 23]. Following a conceptual framework (Supplementary Fig. 1, in the supplementary material), we selected variables that could influence both ambient temperature and EBF duration. Specifically, models were adjusted for year and month of the child assessment to capture long-term trends and seasonal patterns in ambient temperature, as well as factors that may influence breastfeeding (e.g. infectious disease dynamics, household routines, and changes in access or demand for health services). Models were also adjusted for the region of residence (North, Northeast, Central-West, Southeast, and South) to capture broad geographic and structural factors that may influence ambient temperature (e.g. longitude, vegetation, and water bodies) and EBF (e.g. health-service structure and cultural practices). Since our study population comprised children from all 5570 Brazilian municipalities and exposure was defined at the municipal level, we adjusted for region of residence rather than municipality to minimize data sparsity and preserve temperature exposure variability.

Other individual or household characteristics, including child’s age and sex and housing conditions, were not included as adjustment covariates in the models. Although these factors may influence breastfeeding practices or thermal comfort, they do not influence weather conditions and therefore were not considered confounders of the association between ambient temperature and EBF.

Potential effect modifiers of the relationship between ambient temperature and EBF included infant age group (0–2 months, 2–4 months, and 4 to <6 months), infant sex (male, female), and socioeconomic profile of the municipality of residence, assessed using the Brazilian Deprivation Index (IBP). The IBP is a measure of material deprivation, reflecting the socioeconomic conditions in different geographic areas across Brazil [24].

### Ethical aspects

The present study was approved by the School of Nutrition (ENUFBA) (reference number 76672823.8.0000.5023). This study was exempt from obtaining informed consent from participants, as it exclusively used de-identified electronic data.

### Statistical analysis

The association between ambient temperature and time to interruption of EBF was assessed using Cox proportional hazards models combined with distributed lag non-linear models (DLNM) [25].

To accommodate the characteristics of EBF data—characterized by irregular observation intervals and the inability to precisely identify the exact day of EBF interruption—the event time was specified within an interval-censoring structure [26]. In this framework, each child was represented by a time interval containing the possible occurrence of the event. Three situations were considered: (i) children who transitioned from EBF = 1 (exclusively breastfed) to EBF = 0 (no longer exclusively breastfed) were defined as interval-censored events, with the lower limit (L) set at the last visit when the child was exclusively breastfed and the upper limit (R) at the first visit without EBF; (ii) children who entered the study already not exclusively breastfed were classified as immediate events, with L = 0 (birth) and R equal to the age at the first visit; and (iii) children who remained exclusively breastfed throughout follow-up were treated as right-censored, with L corresponding to the last observed visit and R = 182 days representing the end of the follow-up period (5 months and 29 days).

A matrix of exposure histories was constructed for the study population using daily mean ambient temperature data at the municipal level. Lags were defined with the R of each follow-up interval designated as lag 0, and exposures from the 6 preceding days (lags 0–6). The 0–6 lag structure was chosen based on evidence indicating that short-term associations between temperature and breastfeeding practices persist within a weekly timeframe [27, 28].

A cross-basis was specified using a natural cubic spline with three internal knots (at the 25th, 75th, and 90th percentiles) in the temperature dimension and a natural cubic spline with two internal knots in the lag dimension. The number and position of knots were guided by the Akaike information criterion (Supplementary Table 2, in the supplementary material).

Hazard ratios (HRs) and 95% confidence intervals (CIs) were estimated for cold (1st percentile) and heat (99th percentile), using the median temperature as the reference value. The reference value (median) was calculated based on the temperature distribution over the 0–6 lag period.

All models were adjusted for the region of residence (North, Northeast, Central-West, Southeast, South), the year and month of the child’s assessment, and absolute humidity. Stratified analyses were conducted by infant age group (0–2, 2–4, and 4 to <6 months), infant sex (male, female), and IBP (low, medium, high).

Several sensitivity analyses were performed to assess the robustness of the findings to alternative modeling assumptions and exposure-window definitions. First, to account for the possibility of an association when EBF interruption occurred earlier than the 0–6-day window preceding the visit, we repeated the main analysis using longer lag periods: 0–13 and 0–20 days. Second, to address uncertainty about the relevant exposure window and heterogeneity in the interval between visits, we performed additional analyses restricted to infants with intervals of 14 and 21 days between visits and defined exposure as the average daily temperature across the entire interval. Third, we evaluated alternative cross-basis specifications by varying the number and placement of knots. Fourth, given the lack of consensus regarding the role of humidity as a confounder in temperature–health associations, we fitted models without adjustment for absolute humidity. Finally, we fitted models using strata for region of residence to provide an alternative control for regional differences in climate, socioeconomic context, healthcare access, and breastfeeding practices.

All statistical analyses were conducted in R software, version 4.1.2, using the dlnm and survival packages.

## Results

**Figure 2:**
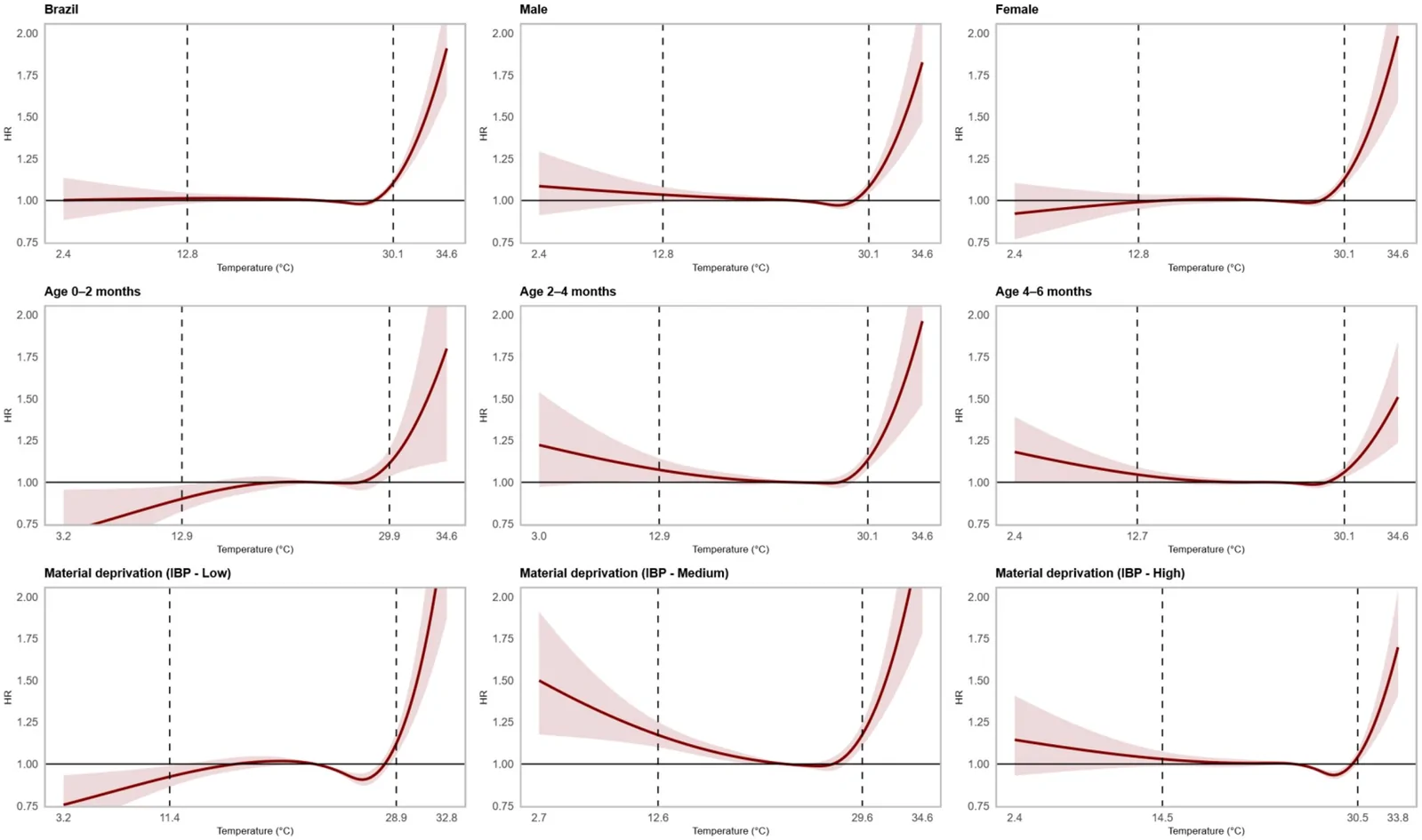
Association between ambient temperature and interruption of EBF among infants aged 0–5 months in Brazil and population subgroups (child sex, child age, and municipal material deprivation), 2015–2019. The shaded areas represent the 95% confidence intervals, and the dotted lines indicate the 1st (cold) and 99th (heat) percentiles of the temperature distribution.

A total of 397 239 children aged 0 to <6 months were included in the study after applying the exclusion criteria (Fig. 1—flowchart). The prevalence of EBF was 43.3%, with a median duration of 2 months. Characteristics of the study population are presented in Table 1 and descriptive statistics for daily mean temperature are presented in Supplementary Table 1, in the supplementary material. Figure 2 shows the temperature–response curves for the risk of EBF interruption for Brazil and the population subgroups (child sex and age, and municipal material deprivation). At the national level, the curve exhibited a J-shaped pattern, with a flat risk at low and moderate temperatures followed by a sharp increase in risk at higher temperatures. Subgroup curves generally showed similar patterns, although some slight decrease in risk (infants aged 0–2 months and low-deprivation municipalities) or slight increase (infants aged 2–4 months and medium-deprivation municipalities) was observed at lower temperatures.

**Table 1:** Characteristics of study population according to breastfeeding practices (SISVAN, Brazil, 2015–2019).

	Variable	Total *n* (%)	EBF *n* (%)	Non-EBF *n* (%)	*P*-value
Child sex	Male	202 303 (50.93)	112 084 (55.40)	90 219 (44.60)	<.0000
	Female	194 936 (49.07)	111 085 (56.98)	83 851 (43.02)	
Age (months)	0 to < 2	179 805 (45.26)	130 099 (72.36)	49 706 (27.64)	<.0000
	2 to < 4	123 990 (31.21)	65 469 (52.80)	58 521 (47.20)	
	4 to < 6	93 444 (23.52)	27 601 (29.54)	65 843 (70.46)	
Region of residence	North	20 181 (5.08)	11 869 (58.81)	8312 (41.19)	<.0000
	Northeast	70 408 (17.72)	31 550 (44.81)	38 858 (55.19)	
	Central-West	22 132 (5.57)	13 367 (60.40)	8765 (39.60)	
	Southeast	234 477 (59.03)	137 604 (58.68)	96 873 (41.32)	
	South	50 041 (12.60)	28 779 (57.51)	21 262 (42.49)	
Municipal material deprivation (IBP)	Low (Q1+Q2)	118 266 (29.77)	72 424 (61.24)	45 842 (38.76)	<.0000
	Medium (Q3)	98 759 (24.86)	56 397 (57.11)	42 362 (42.89)	
	High (Q4+Q5)	180 214 (45.37)	94 347 (52.35)	85 867 (47.65)	

All variables showed a *P*-value <.000 (2.2 × 10^−16^) in the chi-square test.

Table 2 presents the cumulative HRs and 95% CIs for the interruption of EBF for cold (1st percentile vs. median) and hot (99th percentile vs. median) temperatures. Nationally, low temperatures were not significantly associated with EBF interruption (HR 1.01, 95% CI 0.98–1.05), whereas heat exposure was associated with a 10% higher risk (HR 1.10, 95% CI 1.08–1.14).

**Table 2:** HRs and 95% CIs associated with cold (1st percentile vs. median) and heat (99th percentile vs. median) for interruption of EBF among infants aged 0–5 months in Brazil (2015–2019), overall and by population subgroups.

	Subgroup	HR (95% CI)^Cold^	HR (95% CI)^Heat^
Brazil	All	1.01 (0.98; 1.05)	1.10 (1.08; 1.14)
Age	Age [0–2)	0.90 (0.83; 0.96)	1.10 (1.03; 1.20)
	Age [2–4)	1.07 (1.01; 1.14)	1.13 (1.08; 1.19)
	Age [4–6)	1.05 (1.00; 1.09)	1.06 (1.02; 1.10)
Sex	Male	1.03 (0.99; 1.06)	1.08 (1.04; 1.12)
	Female	0.99 (0.94; 1.04)	1.13 (1.09; 1.16)
Municipal material deprivation (IBP)	Low	0.92 (0.86; 0.99)	1.12 (1.06; 1.19)
	Medium	1.17 (1.10; 1.21)	1.18 (1.11; 1.25)
	High	1.03 (0.99; 1.07)	1.04 (1.00; 1.08)

Models were adjusted for region of residence, year and month of the assessment, and absolute humidity.

In age-stratified analyses, cold temperatures were associated with a decreased risk of EBF interruption among infants aged 0–2 months (HR 0.90, 95% CI 0.83–0.96), whereas an increased risk was observed among those aged 2–4 months (HR 1.07, 95% CI 1.01–1.14). In contrast, heat exposure consistently increased the risk of EBF interruption across all age groups.

We found no evidence of sex differences in the association between extreme temperatures and EBF interruption: there was no significant association with cold exposure for either sex, whereas heat exposure was significantly associated with EBF interruption among both male (HR 1.08, 95% CI 1.04–1.12) and female infants (HR 1.13, 95% CI 1.09–1.16).

We also found no consistent evidence of effect modification by material deprivation (IBP). However, some heterogeneity in cold-related estimates was observed across deprivation strata: cold temperatures were associated with reduced risk in low-deprivation areas (HR 0.92, 95% CI 0.86–0.99), increased risk in medium-deprivation areas (HR 1.17, 95% CI 1.10–1.24), and no significant association in high-deprivation areas (HR 1.03, 95% CI 0.99–1.07). Conversely, heat exposure was associated with increased risk of EBF interruption across all IBP strata, low (HR 1.12, 95% CI 1.06–1.19), medium (HR 1.18, 95% CI 1.11–1.25), and high (HR 1.04, 95% CI 1.00–1.08).

Sensitivity analysis results are presented in Supplementary Table 3, in the supplementary material. Overall, the associations were consistent across alternative model specifications and exposure-window definitions, particularly for heat exposure. At the national level, heat-related associations remained similar when longer lag periods were used (lag 0–13: HR 1.10; 95% CI 1.07–1.14; lag 0–20: HR 1.12; 95% CI 1.08–1.15) and were higher in analyses restricted to infants with shorter intervals between visits (≤14 days: HR 1.25; 95% CI 1.08–1.44; ≤21 days: HR 1.23; 95% CI 1.10–1.37). This pattern was generally observed across population subgroups in models using longer lag periods, although estimates were less precise in the restricted analyses. Cold-related associations were more sensitive to the exposure-window definition. At the national level, the association with cold was close to null in the main analysis, but positive associations were observed in models with longer lags (lag 0–13: HR 1.04; 95% CI 1.00–1.08; lag 0–20: HR 1.07; 95% CI 1.03–1.10) and in the analysis restricted to shorter intervals between visits (≤14 days: HR 1.28; 95% CI 1.13–1.45; ≤21 days: HR 1.14; 95% CI 1.03–1.26). The inverse associations observed among infants aged 0–2 months and in municipalities with low material deprivation were attenuated in models with longer lags and were not observed in the restricted analyses. Cold-related associations also showed variability in the model without adjustment for absolute humidity, where statistically significant associations were observed for Brazil (HR 1.09; 95% CI 1.05–1.12) and for the high-deprivation subgroup (HR 1.14; 95% CI 1.10–1.19).

## Discussion

In this nationwide longitudinal study, we found that higher ambient temperature was consistently associated with an increased risk of early interruption of EBF among Brazilian infants. The increased risk associated with heat exposure was similar across infant age groups, child sex, and levels of municipal material deprivation. In contrast, we found no association between cold temperatures and EBF interruption at the national level. However, our findings suggest that cold-related effects may differ across infant age and municipal deprivation levels.

Our results align with emerging literature from previous studies conducted across South Asia, sub-Saharan Africa, and the Middle East, which reported associations between higher ambient temperature or hotter seasons and reduced breastfeeding prevalence or duration [6, 10–12, 29–31]. Nonetheless, much of this earlier evidence relied on seasonal proxies (e.g. summer months), which limit the ability to isolate temperature-specific effects from other seasonal processes unrelated to temperature.

More recent evidence based on direct temperature measurements further supports our findings. Evidence from a sub-Saharan African community suggests that higher daily mean temperatures are associated with shorter breastfeeding duration among children aged 0–13 months and with lower odds of receiving EBF among infants aged 0–3 months [6]. Similarly, a study in a Bangladeshi community reported that higher wet-bulb globe temperatures were associated with decreased daily breastfeeding hours among children under 6 months of age [29]. In addition, a multi-country analysis across 36 low- and middle-income countries found that heatwaves were associated with reduced breastfeeding frequency among infants aged 6–23 months [32].

Several mechanisms may explain the observed heat-related decrease in breastfeeding duration. Higher temperatures may increase infant discomfort, potentially leading caregivers to interpret crying or restlessness as signs of hunger or thirst and to introduce water or other liquids prematurely [23]. Qualitative research has also shown that mothers frequently perceive that infants require additional fluids during hot weather, which can prompt premature introduction of liquids contrary to WHO guidelines [33]. In addition, high ambient temperatures may also affect maternal comfort with breastfeeding, particularly in households with limited cooling devices [7]. Heat may also influence caregiving routines, maternal hydration and nutrition, and perceived milk sufficiency, which often drive early supplementation [7, 22]. Previous evidence indicates that susceptibility to heat exposure varies across the life course and population groups [34, 35]. However, we found no evidence of effect modification in the relationship between heat and EBF by child age and sex, with all strata showing increased risk. One possible explanation is that early infancy (0 to <6 months) may be characterized by broadly similar thermoregulatory and physiological responses, with limited differentiation by sex, resulting in comparable susceptibility across groups. Another possible explanation is that heat-related effects on breastfeeding practices may operate primarily through maternal pathways, such as heat discomfort or beliefs about breastfeeding during hot weather. In other words, this suggests that physiological and behavioral responses to heat affecting breastfeeding are more strongly driven by caregiving dynamics and maternal well-being than by infant sex and age. Nonetheless, empirical data on vulnerability to heat according to infant and maternal characteristics are scarce, and further research is needed to clarify these mechanisms.

We also found no association between cold temperatures and EBF interruption at the national level. This finding differs from a cohort study conducted in Southern Brazil reporting shorter EBF duration among infants born in colder months, a pattern that may reflect the more pronounced seasonal variability and lower winter temperatures characteristic of the South region [13]. Nonetheless, our age-stratified analyses suggest that the EBF association with cold temperatures may vary across early developmental stages. Among infants aged 0–2 months, cold temperatures were associated with a reduced risk of EBF interruption, whereas an opposite association was observed among those aged 2–4 months. Age-related differences in feeding patterns, and maternal perceptions of vulnerability to cold might explain these divergent patterns. For very young infants, colder temperatures may reinforce protective caregiving behaviors, such as spending more time indoors, maintaining closer physical contact, and offering breastfeeding for comfort. As infants reach 2–4 months, increased social contact and transitions in daily routines may overlap with a higher occurrence of respiratory symptoms, potentially interfering with breastfeeding or leading to early supplementation [7]. These factors may contribute to the age-specific patterns observed for cold exposure, in contrast to heat, for which the effects on breastfeeding during early infancy may be more homogeneous.

A similar pattern was observed across deprivation strata, with heat-related estimates showing no significant differences across deprivation levels, in contrast to the heterogeneous patterns observed for low temperatures. Reduced risk in low-deprivation municipalities may reflect conditions that help sustain EBF during colder periods. Conversely, the increased risk observed in medium-deprivation municipalities may reflect settings where socioeconomic vulnerabilities influence caregiving responses to cold temperatures. In high-deprivation areas, the lack of significant associations could reflect limited exposure variation, as these municipalities are predominantly located in the North region, where temperature variability is reduced.

Furthermore, our measure of municipal material deprivation, while a reasonable proxy for socioeconomic status, may insufficiently capture individual or contextual characteristics that may influence breastfeeding responses to extreme temperatures, such as household characteristics (e.g. ventilation, density), urban–rural setting, and maternal working conditions. These factors vary substantially across Brazil and are patterned by longstanding socioeconomic inequalities. Families living in poorly insulated homes or densely populated urban areas may experience higher and more persistent indoor temperatures, increasing thermal discomfort for both mothers and infants. Conversely, families with higher socioeconomic status generally have access to a wider range of adaptive resources to reduce the effects of environmental exposure, as well as more flexible caregiving arrangements and better working conditions. Future research should further examine the mechanisms and modifiers influencing temperature-related breastfeeding outcomes, including caregiving factors and household characteristics.

Our findings should be interpreted in light of some limitations. First, exposures were assigned using municipal-level temperature data, which may not fully capture individual-level temperature exposure, particularly in areas with diverse microclimates or heterogeneous housing conditions. However, this type of exposure misclassification tends to bias estimates toward the null [36]. In addition, we relied on ambient temperature as the primary heat exposure metric, which may not fully capture the physiological heat burden experienced by lactating mothers and infants. Future studies should explore the use of thermal stress indices (e.g. Universal Thermal Climate Index) to provide a more comprehensive assessment of heat stress and its relationship with breastfeeding outcomes. Third, although SISVAN provides standardized population-based information, it relies on routine primary care visits with varying intervals between appointments. Because the exact day of EBF interruption cannot be observed, we utilized an interval-censoring framework. While the DLNM approach allows us to account for cumulative and delayed exposure over a 0–6 day lag period, uncertainty remains regarding the exact temporal alignment between the modeled temperature window and the actual behavioral transition. Nevertheless, our sensitivity analyses using alternative lag windows (0–13 and 0–20 days) as well as restricting the sample to shorter visit intervals yielded similar results, supporting the robustness of our main findings. Finally, despite adjusting for humidity, long-term trends, and seasonality, residual confounding by unmeasured seasonal factors cannot be fully ruled out.

In conclusion, we found that higher ambient temperatures were associated with increased risk of early interruption of EBF in Brazil, with consistent patterns across infant age groups, sex, and socioeconomic strata. These findings are particularly relevant in the context of climate change in which rising temperatures may compromise optimal breastfeeding practices, one of the most cost-effective interventions for child survival, especially in vulnerable populations.

## Ethics approval

The present study was approved by the School of Nutrition (ENUFBA) (reference number 76672823.8.0000.5023). This study was exempt from obtaining informed consent from participants, as it exclusively used de-identified electronic data.

## Author contributions

R.C.R.S., T.R.C., L.S.S., L.H.L.R., O.T.R., P.C.J., D.D., I.H.S., L.S.V., E.S.P., P.R.F.C., and M.L.B. participated on the conceptualization, formal analysis, writing the original draft, and critically review & editing the intellectual content of the manuscript. All authors approved the final submitted version of this manuscript and accepted accountability for all aspects of the work.

## Supplementary Material

dyag205_Supplementary_Data

## Data Availability

The data supporting the findings of this study are available from the Centre for Data and Knowledge Integration for Health (CIDACS), Oswaldo Cruz Foundation. Due to ethical and privacy restrictions, these data are not publicly available. Access may be requested and granted following CIDACS’ data access procedures (https://cidacs.bahia.fiocruz.br/).
