# Supplementary material for: Ambient temperature and exclusive breastfeeding: a nationwide cohort study in Brazil
Source: Int J Epidemiol. 2026 Sep 17;55(5):dyag205. doi: 10.1093/ije/dyag205 (PMC13585394; doi:10.1093/ije/dyag205)
Supplement: Supplementary file 1 [file dyag205_supplementary_data.docx]

**Supplementary material**

**Title:** Ambient temperature and exclusive breast-feeding: A nationwide cohort study in Brazil

**Table of contents**

[**Figure S1**: Conceptual framework	2](#_Toc231943851)

[**Table S1**: Descriptive statistics of daily mean temperature data (°C)	3](#_Toc231943852)

[**Table S2** Comparison of alternative temperature cross-basis specifications based on the Akaike Information Criterion	4](#_Toc231943853)

[**Table S3**: Sensitivity analysis	5](#_Toc231943854)

# **Figure S1**: Conceptual framework

**Figure S1**. Causal directed acyclic graph of the relationship between ambient temperature and interruption of exclusive breastfeeding among infants aged 0 to <6 months. The yellow node represents the exposure (ambient temperature), the dark blue node represents the outcome (interruption of exclusive breastfeeding), the light blue nodes represent the potential causes of the outcome, and the red nodes represent the common causes of both the exposure and the outcome. Red arrows indicate potential backdoor paths linking ambient temperature to exclusive breastfeeding (Panel A). As shown in Panel B, the sufficient adjustment set for estimating the total effect of ambient temperature on interruption of exclusive breastfeeding includes region of residence, month of assessment, year of assessment, and absolute humidity. Conditioning on these variables blocks all backdoor (bias) paths implied by the model.

# **Table S1**: Descriptive statistics of daily mean temperature data (°C)

| Region | Minimum | Mean | Median | IQR | Maximum |
| --- | --- | --- | --- | --- | --- |
| Brazil | 2.4 | 23.4 | 23.9 | 5.2 | 34.6 |
| Central-West | 7.8 | 24.6 | 25.2 | 4.10 | 33.9 |
| Northeast | 15.2 | 26.5 | 26.8 | 2.8 | 33.8 |
| North | 16.5 | 27.8 | 27.9 | 1.60 | 34.6 |
| Southeast | 6.2 | 22.6 | 23.0 | 4.5 | 32.8 |
| South | 2.4 | 20.4 | 20.8 | 6.2 | 32.8 |

IQR: interquartile range.

# **Table S2** Comparison of alternative temperature cross-basis specifications based on the Akaike Information Criterion

| Model | Temperature knots | ΔAIC |
| --- | --- | --- |
| Main model | 25th, 75th, and 90th percentiles | 0.00 |
| Model 2 | 10th, 75th, and 90th percentiles | 0.91 |
| Model 3 | 10th and 90th percentiles | 25.03 |
| Model 4 | 25th and 75th percentiles | 25.80 |

Note: AIC = Akaike Information Criterion. ΔAIC was calculated as the difference between each model’s AIC and the lowest AIC.

# **Table S3**: Sensitivity analysis

**Table S3:** Hazard ratios and 95% confidence intervals associated with cold (1st percentile) and heat (99th percentile) for interruption of exclusive breastfeeding among infants aged 0 to <6 months in Brazil (2015–2019), overall and by population subgroups.

| **Subgroup** | **Model** | **HR (95% CI) _Cold_** | **HR (95% CI) _Heat_** |
| --- | --- | --- | --- |
| Brazil | Main model | 1.01 (0.98 ; 1.05) | 1.10 (1.08 ; 1.14) |
|  | Model using 14 days lag | 1.04 (1.00 ; 1.08) | 1.10 (1.07 ; 1.14) |
|  | Model using 21 days lag | 1.07 (1.03 ; 1.10) | 1.12 (1.08 ; 1.15) |
|  | Restricted analysis: <= 14 days between visits | 1.28 (1.13 ; 1.45) | 1.25 (1.08 ; 1.44) |
|  | Restricted analysis: <= 21 days between visits | 1.14 (1.03 ; 1.26) | 1.23 (1.10 ; 1.37) |
|  | Temperature with knots p10, p75, p90 | 1.01 (0.98 ; 1.05) | 1.11 (1.08 ; 1.14) |
|  | Temperature with knots p10, p90 | 1.01 (0.98 ; 1.04) | 1.08 (1.06 ; 1.11) |
|  | Temperature with knots p25, p75 | 1.01 (0.98 ; 1.04) | 1.08 (1.06 ; 1.11) |
|  | Model without adjustment for humidity | 1.09 (1.05 ; 1.12) | 1.10 (1.07 ; 1.13) |
|  | Model using strata for region of residence | 1.00 (0.97 ; 1.04) | 1.10 (1.07 ; 1.13) |
| Age [0 - 2) | Main model | 0.90 (0.83 ; 0.96) | 1.10 (1.03 ; 1.20) |
|  | Model using 14 days lag | 0.94 (0.85 ; 1.03) | 1.12 (1.03 ; 1.21) |
|  | Model using 21 days lag | 0.97 (0.88 ; 1.07) | 1.13 (1.04 ; 1.23) |
|  | Restricted analysis: <= 14 days between visits | 1.23 (1.06 ; 1.43) | 1.10 (0.87 ; 1.40) |
|  | Restricted analysis: <= 21 days between visits | 1.10 (0.97 ; 1.25) | 1.22 (1.02 ; 1.46) |
|  | Temperature with knots p10, p75, p90 | 0.90 (0.82 ; 0.98) | 1.11 (1.03 ; 1.20) |
|  | Temperature with knots p10, p90 | 0.90 (0.83 ; 0.99) | 1.09 (1.02 ; 1.18) |
|  | Temperature with knots p25, p75 | 0.90 (0.83 ; 0.99) | 1.10 (1.02 ; 1.19) |
|  | Model without adjustment for humidity | 1.00 (0.92 ; 1.09) | 1.09 (1.01 ; 1.18) |
|  | Model using strata for region of residence | 0.90 (0.82 ; 0.98) | 1.11 (1.03 ; 1.20) |
| Age [2 - 4) | Main model | 1.07 (1.01 ; 1.14) | 1.13 (1.08 ; 1.19) |
|  | Model using 14 days lag | 1.10 (1.03 ; 1.18) | 1.13 (1.07 ; 1.19) |
|  | Model using 21 days lag | 1.12 (1.04 ; 1.20) | 1.15 (1.09 ; 1.21) |
|  | Restricted analysis: <= 14 days between visits | 0.92 (0.69 ; 1.23) | 1.14 (0.92 ; 1.40) |
|  | Restricted analysis: <= 21 days between visits | 1.06 (0.84 ; 1.34) | 1.03 (0.88 ; 1.21) |
|  | Temperature with knots p10, p75, p90 | 1.07 (1.01 ; 1.14) | 1.14 (1.08 ; 1.20) |
|  | Temperature with knots p10, p90 | 1.07 (1.01 ; 1.14) | 1.12 (1.07 ; 1.18) |
|  | Temperature with knots p25, p75 | 1.07 (1.01 ; 1.14) | 1.12 (1.07 ; 1.18) |
|  | Model without adjustment for humidity | 1.12 (1.05 ; 1.19) | 1.13 (1.07 ; 1.19) |
|  | Model using strata for region of residence | 1.06 (1.00 ; 1.13) | 1.14 (1.08 ; 1.19) |
| Age [4 - 6] | Main model | 1.05 (1.00 ; 1.09) | 1.06 (1.02 ; 1.10) |
|  | Model using 14 days lag | 1.06 (1.01 ; 1.11) | 1.06 (1.02 ; 1.10) |
|  | Model using 21 days lag | 1.08 (1.03 ; 1.13) | 1.07 (1.03 ; 1.11) |
|  | Restricted analysis: <= 14 days between visits | 1.34 (1.09 ; 1.64) | 1.33 (1.07 ; 1.64) |
|  | Restricted analysis: <= 21 days between visits | 1.13 (0.96 ; 1.33) | 1.26 (1.08 ; 1.48) |
|  | Temperature with knots p10, p75, p90 | 1.04 (1.00 ; 1.09) | 1.06 (1.03 ; 1.10) |
|  | Temperature with knots p10, p90 | 1.04 (1.00 ; 1.09) | 1.05 (1.01 ; 1.09) |
|  | Temperature with knots p25, p75 | 1.04 (1.00 ; 1.09) | 1.05 (1.01 ; 1.08) |
|  | Model without adjustment for humidity | 1.05 (1.01 ; 1.10) | 1.06 (1.02 ; 1.10) |
|  | Model using strata for region of residence | 1.04 (1.00 ; 1.09) | 1.06 (1.02 ; 1.10) |
| Male | Main model | 1.03 (0.99 ; 1.06) | 1.08 (1.04 ; 1.12) |
|  | Model using 14 days lag | 1.06 (1.01 ; 1.11) | 1.08 (1.03 ; 1.12) |
|  | Model using 21 days lag | 1.08 (1.03 ; 1.14) | 1.09 (1.04 ; 1.14) |
|  | Restricted analysis: <= 14 days between visits | 1.34 (1.11 ; 1.61) | 1.17 (0.98 ; 1.40) |
|  | Restricted analysis: <= 21 days between visits | 1.09 (0.96 ; 1.24) | 1.23 (1.06 ; 1.44) |
|  | Temperature with knots p10, p75, p90 | 1.03 (0.99 ; 1.08) | 1.08 (1.04 ; 1.12) |
|  | Temperature with knots p10, p90 | 1.03 (0.99 ; 1.08) | 1.06 (1.02 ; 1.10) |
|  | Temperature with knots p25, p75 | 1.03 (0.99 ; 1.08) | 1.06 (1.02 ; 1.10) |
|  | Model without adjustment for humidity | 1.03 (0.99 ; 1.08) | 1.08 (1.04 ; 1.12) |
|  | Model using strata for region of residence | 0.98 (0.94 ; 1.03) | 1.13 (1.08 ; 1.17) |
| Female | Main model | 0.99 (0.94 ; 1.04) | 1.13 (1.09 ; 1.16) |
|  | Model using 14 days lag | 1.02 (0.97 ; 1.07) | 1.13 (1.09 ; 1.18) |
|  | Model using 21 days lag | 1.05 (0.99 ; 1.10) | 1.14 (1.10 ; 1.20) |
|  | Restricted analysis: <= 14 days between visits | 1.34 (1.11 ; 1.61) | 1.17 (0.98 ; 1.40) |
|  | Restricted analysis: <= 21 days between visits | 1.19 (1.02 ; 1.38) | 1.19 (1.04 ; 1.36) |
|  | Temperature with knots p10, p75, p90 | 0.99 (0.94 ; 1.04) | 1.13 (1.09 ; 1.18) |
|  | Temperature with knots p10, p90 | 0.99 (0.94 ; 1.03) | 1.11 (1.07 ; 1.15) |
|  | Temperature with knots p25, p75 | 0.99 (0.94 ; 1.04) | 1.11 (1.07 ; 1.15) |
|  | Model without adjustment for humidity | 0.99 (0.94 ; 1.04) | 1.13 (1.09 ; 1.18) |
|  | Model using strata for region of residence | 1.03 (0.98 ; 1.07) | 1.08 (1.04 ; 1.12) |
| IBP - Low | Main model | 0.92 (0.86 ; 0.99) | 1.12 (1.06 ; 1.19) |
|  | Model using 14 days lag | 0.95 (0.88 ; 1.02) | 1.11 (1.04 ; 1.19) |
|  | Model using 21 days lag | 0.98 (0.91 ; 1.06) | 1.17 (1.08 ; 1.26) |
|  | Restricted analysis: <= 14 days between visits | 1.10 (0.93 ; 1.31) | 1.51 (1.27 ; 1.80) |
|  | Restricted analysis: <= 21 days between visits | 1.05 (0.92 ; 1.20) | 1.57 (1.35 ; 1.83) |
|  | Temperature with knots p10, p75, p90 | 0.92 (0.86 ; 0.99) | 1.12 (1.06 ; 1.20) |
|  | Temperature with knots p10, p90 | 0.92 (0.85 ; 0.98) | 1.06 (1.00 ; 1.13) |
|  | Temperature with knots p25, p75 | 0.92 (0.86 ; 0.99) | 1.06 (1.00 ; 1.13) |
|  | Model without adjustment for humidity | 0.97 (0.91 ; 1.03) | 1.10 (1.03 ; 1.16) |
|  | Model using strata for region of residence | 0.93 (0.87 ; 1.00) | 1.11 (1.05 ; 1.18) |
| IBP - Medium | Main model | 1.17 (1.10 ; 1.21) | 1.18 (1.11 ; 1.25) |
|  | Model using 14 days lag | 1.20 (1.12 ; 1.29) | 1.21 (1.13 ; 1.29) |
|  | Model using 21 days lag | 1.22 (1.13 ; 1.31) | 1.25 (1.17 ; 1.34) |
|  | Restricted analysis: <= 14 days between visits | 1.30 (1.04 ; 1.62) | 1.27 (0.93 ; 1.72) |
|  | Restricted analysis: <= 21 days between visits | 1.30 (1.09 ; 1.56) | 1.22 (0.97 ; 1.54) |
|  | Temperature with knots p10, p75, p90 | 1.17 (1.10 ; 1.25) | 1.17 (1.11 ; 1.25) |
|  | Temperature with knots p10, p90 | 1.18 (1.10 ; 1.26) | 1.17 (1.10 ; 1.24) |
|  | Temperature with knots p25, p75 | 1.18 (1.10 ; 1.25) | 1.17 (1.10 ; 1.25) |
|  | Model without adjustment for humidity | 1.15 (1.08 ; 1.22) | 1.19 (1.12 ; 1.26) |
|  | Model using strata for region of residence | 1.16 (1.09 ; 1.24) | 1.19 (1.12 ; 1.26) |
| IBP - High | Main model | 1.03 (0.99 ; 1.07) | 1.04 (1.00 ; 1.08) |
|  | Model using 14 days lag | 1.05 (1.00 ; 1.09) | 1.04 (1.00 ; 1.08) |
|  | Model using 21 days lag | 1.06 (1.01 ; 1.11) | 1.05 (1.00 ; 1.09) |
|  | Restricted analysis: <= 14 days between visits | 1.12 (0.95 ; 1.32) | 1.06 (0.85 ; 1.33) |
|  | Restricted analysis: <= 21 days between visits | 0.98 (0.86 ; 1.11) | 1.17 (0.99 ; 1.38) |
|  | Temperature with knots p10, p75, p90 | 1.03 (0.99 ; 1.08) | 1.05 (1.01 ; 1.09) |
|  | Temperature with knots p10, p90 | 1.03 (0.99 ; 1.08) | 1.00 (0.97 ; 1.04) |
|  | Temperature with knots p25, p75 | 1.03 (0.99 ; 1.08) | 1.00 (0.97 ; 1.04) |
|  | Model without adjustment for humidity | 1.14 (1.10 ; 1.19) | 1.05 (1.02 ; 1.10) |
|  | Model using strata for region of residence | 1.02 (0.98 ; 1.06) | 1.04 (1.00 ; 1.08) |

Subgroups include: infant age groups (Age [0–2): 0 to <2 months; Age [2–4): 2 to <4 months; Age [4–6]: 4 to <6 months), child sex (Male; Female), and municipal material deprivation assessed by the Brazilian Deprivation Index (IBP – Low: quintiles Q1+Q2; Medium: quintile Q3; High: quintiles Q4+Q5). Main model: cross-basis using a natural cubic spline with knots at the 25th, 75th, and 90th percentiles in the temperature dimension and two internal knots in the 0–6 day lag dimension, using the median temperature as the reference. Sensitivity analyses included models using longer lag periods of 0–13 and 0–20 days; restricted analyses including only infants with intervals of ≤14 and ≤21 days between visits, with exposure defined as the average daily temperature across the interval; alternative temperature cross-basis specifications with knots placed at the 25th and 75th percentiles, the 10th and 90th percentiles, and the 10th, 75th, and 90th percentiles; a model without adjustment for absolute humidity; and a model using strata for region of residence.
